# Body Mass Index Trajectories Preceding Incident Mild Cognitive Impairment and Dementia

**DOI:** 10.1001/jamapsychiatry.2022.3446

**Published:** 2022-10-26

**Authors:** Jie Guo, Jiao Wang, Abigail Dove, Hui Chen, Changzheng Yuan, David A. Bennett, Weili Xu

**Affiliations:** 1 Aging Research Center, Department of Neurobiology, Care Sciences and Society, Karolinska Institutet and Stockholm University, Stockholm, Sweden; 2 Department of Epidemiology and Biostatistics, School of Public Health, Tianjin Medical University, Tianjin, China; 3 Tianjin Key Laboratory of Environment, Nutrition and Public Health, Tianjin, China; 4 Center for International Collaborative Research on Environment, Nutrition, and Public Health, Tianjin, China; 5 School of Public Health, Zhejiang University School of Medicine, Hangzhou, China; 6 Department of Nutrition, Harvard T.H. Chan School of Public Health, Boston, Massachusetts; 7 Rush Alzheimer’s Disease Center, Rush University Medical Center, Chicago, Illinois

## Abstract

**Importance:**

Body mass index (BMI) trajectories before the onset of mild cognitive impairment (MCI) and during the progression from MCI to dementia remain unclear.

**Objective:**

To assess the long-term BMI trajectories preceding incident MCI and dementia and explore whether they are associated with brain pathologies.

**Design, Setting, and Participants:**

The Rush Memory and Aging Project (MAP) was an ongoing community-based cohort study. This study included cognitively intact participants aged 60 to 90 years at baseline with annual follow-up from October 1997 to December 2020 (maximum follow-up of 22 years). During the follow-up, participants underwent brain autopsies. Data were analyzed from August 2021 to February 2022 using mixed-effect models.

**Exposures:**

BMI was calculated using height and weight measured at baseline and follow-ups.

**Main Outcomes and Measures:**

Incident MCI and dementia were diagnosed following standard criteria. Neuropathological assessments (including global Alzheimer disease and vascular pathology) were performed for autopsies.

**Results:**

A total of 1390 participants (mean [SD] age, 78.4 [6.5] years; 1063 female [76.5%]) were included in the study. In the analysis of BMI trajectories before MCI (n = 939), during the follow-up (median [IQR] duration, 6 [3-9] years), 371 participants (39.5%) developed MCI, of whom 88 (23.7%) progressed to dementia. Those who developed MCI were older (mean [SD] age, 79.6 [5.9] years vs 76.9 [6.6] years), consumed less alcohol (median [IQR] consumption, 0 [0-5.8] g/day vs 1.1 [0-6.9] g/day), had a lower BMI (mean [SD], 27.2 [4.9] vs 28.2 [5.9]), and were more likely to be apolipoprotein E (*APOE*) ε4 carriers (89 of 371 [24.0%] vs 98 of 568 [17.3%]) compared with those who remained cognitively intact over follow-up. Those who developed dementia were older (mean [SD] age, 81.0 [5.2] years vs 79.1 [6.0] years), had a lower level of physical activity (median [IQR] activity, 1.0 [0-2.5] h/week vs 1.8 [0.2-3.8] h/week), and were more likely to be *APOE* ε4 carriers than those who were dementia-free (33 of 88 [37.5%] vs 56 of 283 [19.8%]). Compared with participants who remained cognitively intact, in those with incident MCI, BMI tended to decline earlier and faster. From 7 years before diagnosis, people with incident MCI had an associated significantly lower BMI (mean difference, −0.96; 95% CI, −1.85 to −0.07) than those who were cognitively intact. Among people with incident MCI, the slopes of BMI decline did not differ significantly between those who did and did not develop dementia (β, −0.03; 95% CI, −0.21 to 0.15). In the analysis of BMI trajectories before autopsy (n = 358), BMI was associated with a faster declination among participants with a high burden of global Alzheimer disease pathology (β for pathology × time highest vs lowest tertile, −0.14; 95% CI, −0.26 to −0.02) or vascular pathology (β for pathology × time^2^ highest vs lowest tertile, 0.02; 95% CI, 0-0.05).

**Conclusions and Relevance:**

Results of this cohort study suggest that among cognitively intact people, significantly lower BMI occurs beginning approximately 7 years before MCI diagnosis. After MCI diagnosis, BMI declines at the same pace in people who develop dementia and those who do not. High brain pathologies may underly the BMI decline preceding dementing disorders.

## Key Points

**Question:**

What are the body mass index (BMI) trajectories before incidence of mild cognitive impairment (MCI) and dementia, and are these trajectories associated with brain pathologies?

**Findings:**

In this cohort study including 1390 cognitively intact older adults, BMI trajectory of those with incident MCI deviated from that of cognitively intact individuals, showing an association with a lower BMI near MCI diagnosis. After MCI diagnosis, BMI declined at a similar pace between incident dementia and dementia-free participants; high Alzheimer disease pathology and cerebrovascular pathology may underly the BMI decline preceding dementing disorders.

**Meaning:**

Monitoring and managing BMI change may have important implications for identifying and preventing cognitive impairment among older adults.

## Teaser

This cohort study assesses body mass index trajectories before incidence of mild cognitive impairment and their association with brain pathologies.

## Introduction

Dementia affects approximately 55 million people worldwide and has emerged as one of the major causes of disability and mortality among older people.^1^ Mild cognitive impairment (MCI), a prodromal phase of dementia and a transitional state between normal cognition and dementia, affects 16% to 20% older adults,^2^ and more than one-third of individuals with MCI go on to develop dementia over 5 or more years.^3^ Therefore, identifying risk factors for MCI may help to slow or prevent the development of dementia.

High body mass index (BMI) in midlife has been established as a modifiable risk factor for both MCI^4,5^ and dementia.^6^ However, the associations between late-life BMI and cognitive health remain unclear. A few studies have reported that high BMI in late life is associated with dementia risk,^7,8^ but others have shown that late-life obesity is either not significantly related to dementia^9,10,11^ or associated with a decreased risk of MCI,^4,5^ dementia,^12,13,14^ and the progression from MCI to dementia.^15,16,17^ These conflicting findings may be attributable to reverse causation because of weight loss preceding dementia.^18^

Clinical diagnosis of dementia is preceded by a decades-long preclinical and prodromal phase, which may lead to weight loss and a subsequent lower BMI.^19,20^ A few studies have investigated long-term BMI trajectories before the diagnosis of dementia,^19,21^ reporting that BMI level in older adults (who later developed dementia) became significantly lower than that in dementia-free participants starting from 2 to 3 years,^19,21^ 6 years,^22^ or up to 10 years^23^ before dementia diagnosis. Using data from the Religious Orders Study, we previously reported that BMI loss was associated with increased risk of Alzheimer dementia and a faster rate of cognitive decline.^14^ However, anchoring the timescale to etiologic stage of dementing disorders instead of the baseline assessment can more explicitly show BMI change. Moreover, evidence on the long-term BMI trajectories before MCI is lacking. Not all people with MCI go on to develop dementia; therefore, MCI is regarded as a reversible state before dementia and provides an opportunity to prevent or delay dementia onset.^24,25^ More studies are warranted to investigate the long-term BMI changes with cognitive phenotypes in aging, from cognitively intact to MCI and its conversion to overt dementia.

Brain pathologies—such as Alzheimer disease (AD) pathologies involving deposits of amyloid plaques—develop decades before the clinical onset of dementia^26^ and may play a role in weight change, given their associations with olfactory dysfunction, reduced appetite, and disrupted energy homeostasis.^27,28^ The mechanisms underlying weight loss in the preclinical and prodromal phase of dementia are not well understood. We previously reported an association of AD pathology with the average BMI proximate to death in the Religious Orders Study.^29^ Here, we extended our prior work by exploring whether BMI trajectories differ according to brain pathology burden.

In the present study, we aimed to (1) examine BMI trajectories preceding the transition from normal cognition to MCI and from MCI to dementia, and (2) explore the association between BMI trajectories and brain pathologies using data from a long-term community-based longitudinal cohort study.

## Methods

### Study Design and Participants

This study was approved by an institutional review board of Rush University Medical Center and is consistent with the ethical standards in the 1964 Declaration of Helsinki and its later amendments. Prior to enrollment, all participants provided informed consent and signed an Anatomical Gift Act for organ donation. All participants signed a repository consent allowing their data to be shared. The Rush Memory and Aging Project (MAP) was an ongoing longitudinal cohort study investigating risk factors for common chronic neurodegenerative conditions in older adults.^30^ Study participants were recruited from continuous care retirement communities, senior and subsidized housing, church groups, social service agencies, and individual homes in northeastern Illinois and the Chicago area.^30^ All participants underwent a comprehensive clinical evaluation, neurological examination, and extensive cognitive tests at enrollment and at follow-up visits thereafter.^30^ Race and ethnicity in this study were required by the US National Institutes of Health (funder of the Rush MAP) and were collected by self-report (eAppendix 1 in the Supplement). This study followed the Strengthening the Reporting of Observational Studies in Epidemiology (STROBE) reporting guidelines.

Between October 1997 and December 2020, MAP enrolled 2192 participants who were annually followed up for up to 22 years. Participants aged 60 to 90 years who were cognitively intact at baseline were included. Participants who developed dementia without a previous diagnosis of MCI and those with fewer than 3 repeated BMI measurements were excluded from the study. Information on sociodemographic factors (age, sex, education), lifestyle factors, diabetes, vascular diseases, and apolipoprotein E (*APOE*) gene variant was collected at study entry. Details about the data collection are described in eAppendix 1 in the Supplement.

### Assessment of BMI

Weight and height were measured at study entry and during annual follow-up visits. Measurements were taken by a trained technician blinded to previously collected data, and participants wore light clothing and no shoes. BMI was calculated as weight in kilograms divided by height in meters squared.

### Assessment of Dementia and MCI

Clinical diagnoses of dementia and MCI were assessed using a uniform, structured process, including computer scoring of cognitive tests, clinical judgment by a neuropsychologist, and diagnostic classification by a clinician.^31,32^ Dementia was diagnosed based on the criteria of the joint working group of the National Institute of Neurological and Communicative Disorders and Stroke and the Alzheimer Disease and Related Disorders Association.^31^ A diagnosis of MCI was rendered for participants who did not meet the criteria for dementia but were judged by a neuropsychologist to have objective impairment in cognitive function, based on data from cognitive tests and considering age and education level.^32,33^

### Assessment of Brain Pathologies

Global AD pathology burden is a quantitative summary of AD pathology derived from counts of 3 AD pathologies: neuritic plaques, diffuse plaques, and neurofibrillary tangles.^34^ We further tertiled global AD pathology. The cerebral vascular disease pathology was tertiled based on the sum of 3 pathologies: atherosclerosis, arteriolosclerosis, and cerebral amyloid angiopathy, and each of them was scored from 0 to 3.^35^

### Statistical Analysis

BMI trajectories were examined using a backward timescale, ie, year 0 was the year of MCI diagnosis or the year corresponding to the end of follow-up (for participants who remained cognitively intact). Mixed-effect models were used to determine the retrospective BMI trajectories. MCI status, time, time^2^, and their interaction were included in the model to test the differences in BMI trajectories by MCI status (coded as 1 for incident MCI and 0 for cognitively intact). The random effect included random intercept and slope to reflect individual differences in both BMI at year 0 and BMI change over time. The difference in BMI between cognitively intact participants and those who developed MCI was estimated for each year preceding year 0, with a negative value indicating lower BMI among the MCI group. All analyses were adjusted for age at time 0, sex, and education and the interaction terms between these variables and time when the *P* value for interactions was <.05. Similarly, we analyzed BMI trajectories before dementia diagnosis among participants with incident MCI, using the year of incident dementia or the last follow-up as year 0. The reference group was participants with incident MCI but not developing dementia during the follow-up. To explore the potential role of brain pathologies in the BMI change in lead-up to dementia, we examined BMI trajectories by global AD and vascular pathologies among dementia-free participants, using the year of death as year 0. To take full advantage of the longitudinal data set, we included all the time points with BMI values. This resulted in a maximum backward timescale of 18 years for MCI, 10 years for dementia after MCI, and 15 years for death.

Supplementary analyses were described in the eAppendix 2 in the Supplement. Statistical analyses were performed using SAS, version 9.4 (SAS Institute). All *P* values were 2-sided, and we defined statistical significance as *P* < .05. For multiple testing and comparisons of differences in estimated BMI, we used a simulation-based approach combined with a step-down fashion to calculate adjusted *P* values and CIs.^36^ Data were analyzed from August 2021 to February 2022.

## Results

### Characteristics of the Study Population

Of the 1390 participants (mean [SD] age, 78.4 [6.5] years), 1063 were women (76.5%); 327 were men (23.5%); and 7 (0.5%) were American Indian or Alaska Native, 61 (4.4%) were Black or African American, 1190 (85.6%) were White, 7 (0.5%) reported other race or ethnicity, and 125 (9.0%) preferred not to answer. We further excluded 22 participants who developed dementia without a previous diagnosis of MCI and 429 participants with fewer than 3 repeated BMI measurements. This left a study population of 939 cognitively intact individuals. Over the follow-up period (median [IQR] duration, 6 [3-9] years), 371 participants (39.5%) developed incident MCI, 88 (23.7%) of whom developed dementia. During the follow-up, a total of 520 participants died and underwent brain autopsy. Of these individuals, 358 who did not have incident dementia before death were included in the analyses of predeath BMI trajectories (eFigure 1 in the Supplement). Of the 939 cognitively intact participants at baseline, those who developed MCI were older (mean [SD] age, 79.6 [5.9] years vs 76.9 [6.6] years), consumed less alcohol (median [IQR] consumption, 0 [0-5.8] g/day vs 1.1 [0-6.9] g/day), had a lower BMI (mean [SD], 27.2 [4.9] vs 28.2 [5.9]), and were more likely to be *APOE* ε4 carriers (89 of 371 [24.0%] vs 98 of 568 [17.3%]) compared with those who remained cognitively intact over follow-up (Table 1). Among 371 participants with incident MCI, those who developed dementia were older (mean [SD] age, 81.0 [5.2] years vs 79.1 [6.0] years), had a lower level of physical activity (median [IQR] activity, 1.0 [0-2.5] h/week vs 1.8 [0.2-3.8] h/week), and were more likely to be *APOE* ε4 carriers than those who were dementia-free (33 of 88 [37.5%] vs 56 of 283 [19.8%]).

**Table 1.** Baseline Characteristics of the Study Population by Incident Mild Cognitive Impairment (MCI) in the Cognitively Intact Group and by Incident Dementia in the Incident MCI Group

Characteristic	Group, No. (%)			
	Cognitively intact (n = 939)		Incident MCI (n = 371)	
	MCI-free	Incident MCI	Dementia-free	Incident dementia
No.	568	371	283	88
Age, mean (SD), y				
At baseline	76.9 (6.6)	79.6 (5.9)	79.1 (6.0)	81.0 (5.2)
At time 0	83.9 (6.9)	85.3 (6.1)	87.9 (6.1)	90.2 (5.4)
Female	448 (78.9)	285 (76.8)	213 (75.3)	72 (81.8)
Male	120 (21.1)	86 (23.2)	70 (24.7)	16 (18.2)
Education, mean (SD), y	15.1 (3.2)	15.0 (3.3)	15.0 (3.4)	14.9 (2.9)
Smoking				
Never	332 (58.5)	234 (63.1)	173 (61.1)	61 (69.3)
Former/current smoker	236 (41.5)	137 (36.9)	110 (38.9)	27 (30.7)
Alcohol consumption, median (IQR), g/d	1.1 (0-6.9)	0 (0-5.8)	0 (0-5.8)	0 (0-5.8)
Physical activity, median (IQR), h/wk	1.9 (0.3-4.0)	2.2 (0.5-4.3)	1.8 (0.2-3.8)	1.0 (0-2.5)
BMI, mean (SD)^a^	28.2 (5.9)	27.2 (4.9)	27.4 (4.9)	26.5 (4.7)
Diabetes	78 (13.7)	40 (10.8)	31 (11.0)	9 (10.2)
Any vascular diseases^b^	231 (40.7)	142 (38.3)	129 (45.6)	45 (51.1)
Apolipoprotein E ε4 carriers	98 (17.3)	89 (24.0)	56 (19.8)	33 (37.5)

Abbreviation: BMI, body mass index.

^a^ Calculated as weight in kilograms divided by height in meters squared.

^b^ Vascular diseases included claudication, stroke, heart conditions (ie, heart attack or coronary, coronary thrombosis, coronary occlusion, or myocardial infarction), and congestive heart failure.

### BMI Trajectories Before the Development of MCI

**Figure 1.**
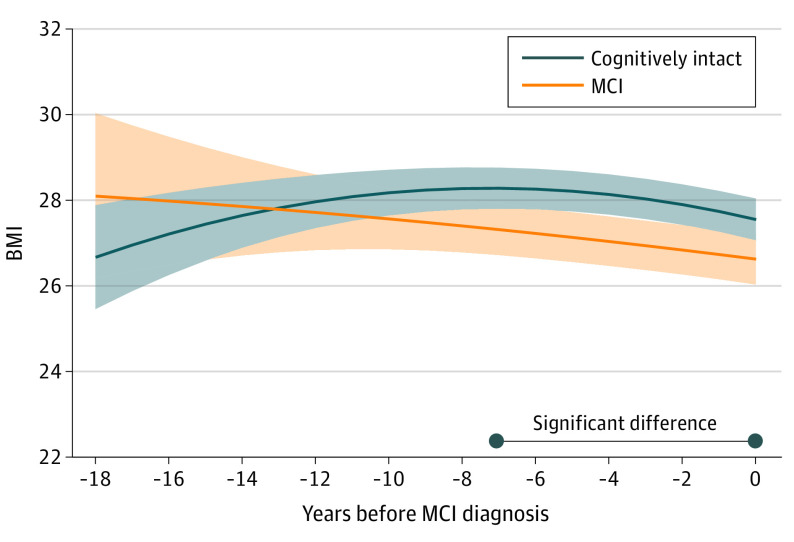
Trajectories of Body Mass Index (BMI) in the 18 Years Before Mild Cognitive Impairment (MCI) Diagnosis The figure represents marginal effects of MCI on trajectories of BMI, adjusted for age at time 0, sex, and education. The band represents the 95% CI of estimated mean BMI. For the multiple testing and comparisons, the significant differences were defined using adjusted *P* values <.05 calculated from a simulation-based approach combined with a step-down fashion.

Figure 1 shows the BMI trajectories for up to 18 years before MCI diagnosis. The trajectories of BMI differed between participants with incident MCI and those who remained cognitively intact, such that those with incident MCI tended to have an earlier associated decline and a more pronounced BMI decline (eTable 1 in the Supplement). BMI level among participants who developed MCI was slightly higher than the estimated level of those who remained cognitively intact from years −18 to −14 (difference in mean BMI at year −18, 1.42; 95% CI, −1.37 to 4.22; at year −14, 0.21; 95% CI, −1.46 to 1.88) (Table 2). BMI level was significantly lower in participants with incident MCI beginning approximately 7 years preceding MCI diagnosis (difference in mean BMI at year −7, −0.96; 95% CI, −1.85 to −0.07).

**Table 2.** Differences in Body Mass Index (BMI) Between Mild Cognitive Impairment (MCI) Cases and Cognitively Intact Ones in the 18 Years Before MCI Diagnosis

Year^a^	No. of cognitively intact participants	No. of MCI	Incident MCI vs cognitively intact	
			Difference, mean of BMI (95% CI)^b^	*P* value
−18	3	1	1.42 (−1.37 to 4.22)	.31
−17	13	2	1.08 (−1.39 to 3.55)	.39
−16	29	5	0.77 (−1.40 to 2.94)	.47
−15	43	10	0.48 (−1.43 to 2.38)	.62
−14	60	16	0.21 (−1.46 to 1.88)	.79
−13	73	18	−0.03 (−1.49 to 1.43)	.96
−12	91	26	−0.25 (−1.54 to 1.05)	.70
−11	105	39	−0.44 (−1.60 to 0.72)	.46
−10	130	46	−0.61 (−1.66 to 0.45)	.25
−9	166	71	−0.75 (−1.73 to 0.23)	.11
−8	212	94	−0.87 (−1.79 to 0.06)	.05
−7	253	124	−0.96 (−1.85 to −0.07)	.03
−6	297	143	−1.03 (−1.90 to −0.15)	.01
−5	356	181	−1.07 (−1.94 to −0.21)	.009
−4	400	218	−1.09 (−1.95 to −0.23)	.007
−3	472	273	−1.08 (−1.94 to −0.23)	.007
−2	529	337	−1.05 (−1.91 to −0.19)	.01
−1	505	329	−1.00 (−1.87 to −0.12)	.02
0	568	371	−0.92 (−1.82 to −0.02)	.04

^a^ Year column designates the years before MCI diagnosis (for participants who developed MCI during the follow-up) and the end of follow-up (for participants who remained cognitively intact).

^b^ Difference in mean was calculated as the mean of BMI in participants with MCI minus that in those cognitively intact. A negative value signifies that BMI was lower in participants with MCI. Model was adjusted for age at time 0, sex, and education.

### BMI Trajectories During MCI Progression to Dementia

**Figure 2.**
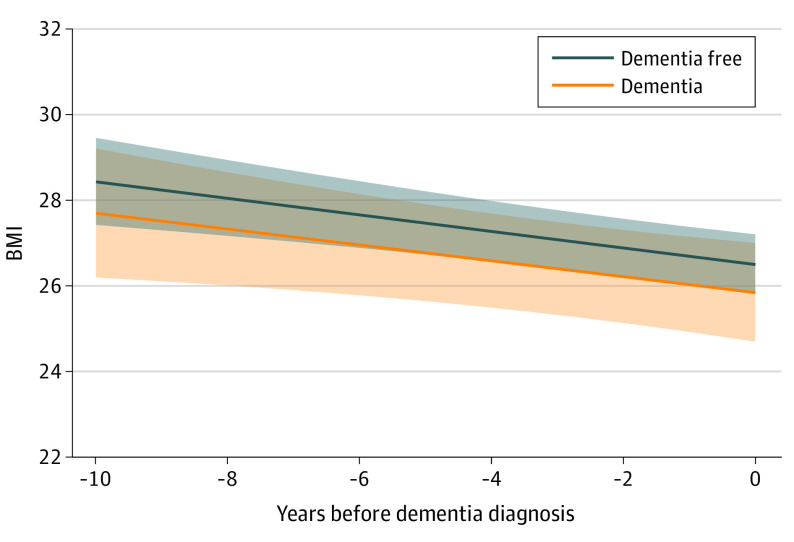
Trajectories of Body Mass Index (BMI) in the 10 Years Before Dementia Diagnosis Among Participants With Incident Mild Cognitive Impairment The figure represents an association of dementia with trajectories of BMI, adjusted for age at time 0, sex, and education. The band represents the 95% CI of estimated mean BMI. The decline in BMI was significant for both dementia-free participants (β, −0.17; 95% CI, −0.32 to −0.02) and those with incident dementia (β, −0.20; 95% CI, −0.30 to −0.10). The difference between their slopes was not statistically significant (dementia vs dementia-free, β, −0.03; 95% CI, −0.21 to 0.15; *P* = .73).

Among participants with incident MCI, BMI declined significantly for both participants who remained dementia free (β, −0.17; 95% CI, −0.32 to −0.02) and those who developed into dementia (β, −0.20; 95% CI, −0.30 to −0.10) for up to 10 years before dementia diagnosis (Figure 2). BMI decline between these 2 groups was not significantly different (β, −0.04; 95% CI, −0.23 to 0.14; *P* = .63) (eTable 2 in the Supplement).

### Trajectories of BMI Before Death Across Brain Pathologies

**Figure 3.**
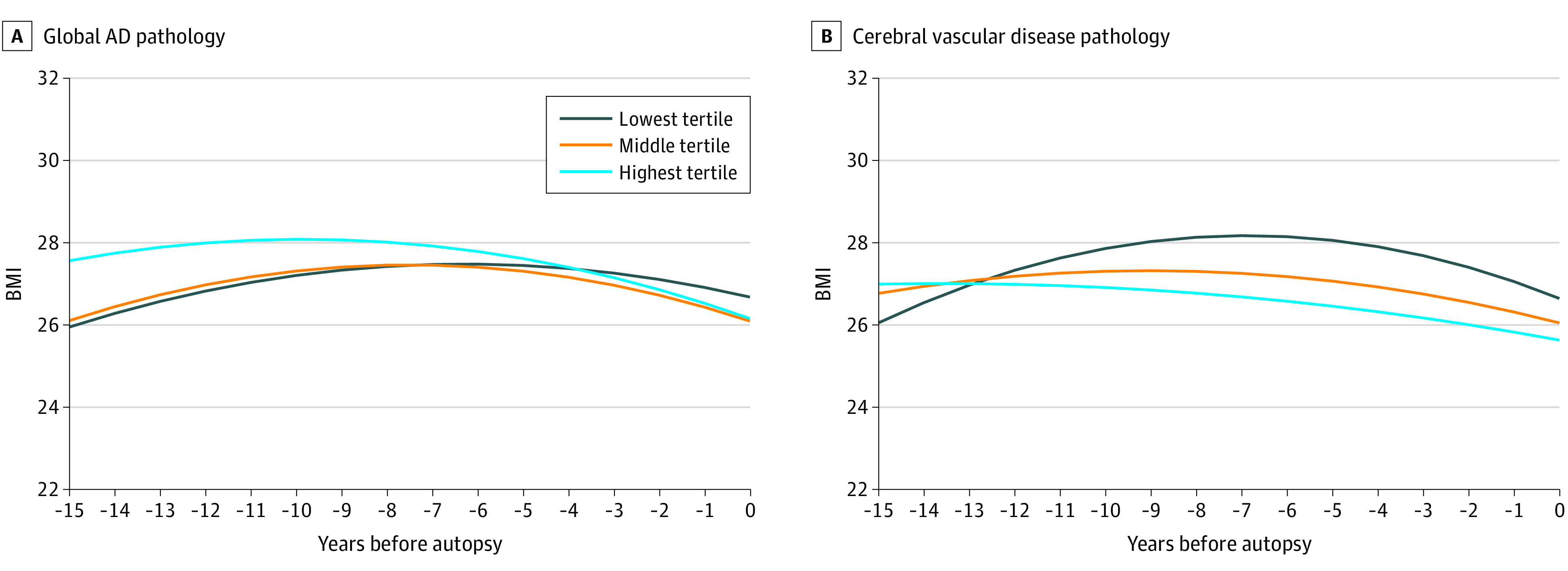
Trajectories of Body Mass Index (BMI) Among Dementia-Free Participants in the 15 Years Before Brain Autopsy, According to Brain Pathologies The figure represents the mean trajectories (solid lines) of BMI across global Alzheimer disease (AD) pathology (A) and cerebral vascular disease pathology (B). Model adjusted for age at time 0, sex, and education. BMI decline before death was faster in the highest tertile of global AD pathology (β for pathology × time highest vs lowest tertile, −0.14; 95% CI, −0.26 to −0.02) and in the highest tertile of cerebral vascular disease pathology (β for pathology × time^2^ highest vs lowest tertile, 0.02; 95% CI, 0-0.05).

Among participants who underwent brain autopsy, the 15-year BMI trajectories before death differed between the highest and lowest tertile burdens of global AD pathology (Figure 3; eTable 3 in the Supplement). BMI decline before death was faster among participants with the higher burden (β for pathology × time highest vs lowest tertile: −0.14; 95% CI, −0.26, −0.02; *P* = .02). BMI trajectories were also significantly different among participants with varied burden of cerebral vascular disease pathology (β for pathology × time^2^ highest vs lowest tertile: 0.02; 95% CI, 0-0.05; *P* = .03).

### Supplementary Results

From years −18 to −7, the decline in BMI was faster in future MCI cases, and per 1 kg/m^2^ decrease per 5-year decrease in BMI was associated with increased MCI (eTables 4 and 5 in the Supplement). The results were largely unchanged after excluding BMI measurements taken within 2 years of death (eFigures 2 and 3 in the Supplement) and after including all participants with at least 1 BMI measurement (results not shown).

## Discussion

In this community-based prospective cohort study of older adults with annually measured BMI, results suggest that (1) BMI declines earlier and a significantly lower BMI starts from the 7 years leading up to MCI diagnosis; (2) after MCI diagnosis, BMI continues to decline at the same pace in people who develop dementia and those who do not; and (3) high burdens of AD pathology and cerebral vascular disease pathology may underly the BMI decline preceding dementing disorders. Our findings point to the BMI decline during the early stage of MCI, which could be used for early detection and prevention of MCI and dementia among older adults.

To date, several studies have explored BMI change by anchoring the diagnosis date of dementia, reporting that BMI decreased faster during the preclinical phase of dementia than in healthy aging, conferring a lower BMI at the time of dementia diagnosis.^19,21,22,23^ However, evidence on the BMI trajectories before the MCI diagnosis is lacking. Assessing BMI trajectory preceding MCI may help to understand whether weight loss is an early sign of dementing disorder even in the absence of cognitive impairment; it may also provide evidence on the inconsistent associations between late-life BMI and MCI. In this study, BMI trajectories among those later diagnosed with MCI differed from those who remained cognitively intact, with an earlier pronounced decline beginning at least 18 years preceding diagnosis and a significantly lower BMI beginning 7 years before diagnosis. Our study suggests that BMI trajectory deviates from that of the natural aging process in the lead-up to the development of MCI, reinforcing the necessity of considering the interval between BMI and MCI assessments. A growing body of literature has explored the association between BMI change and the risk of cognitive impairment,^37,38,39^ showing that weight loss in older adults is associated with an increased risk of MCI.^38^ In our study, a faster BMI decline was associated with a higher risk of MCI, specifically in the 7 to 18 years before diagnosis.

Prospective studies have reported that high BMI is either not significantly associated^17,40^ or only weakly associated^15,16^ with reduced risk of MCI progressing to overt dementia. Moreover, several studies have shown that weight loss is associated with an increased risk of the conversion from MCI to dementia,^15,41,42^ whereas 1 study reported that the significant association only existed among *APOE* ε4 carriers.^43^ In this cohort, BMI values among individuals with MCI who later developed dementia were not significantly different from those who were dementia free, and the declines in BMI between these 2 groups were almost parallel with each other. The discrepancy between our study and previous ones may be due to variations in study populations. The mean age of incident MCI in our study was approximately 85 years, in contrast to approximately 75 years in previous studies.^41,42,43^ Very older age (eg, ≥80 years) itself is associated with weight loss, which may dilute the impact due to prodromal dementia.^44^

There is limited evidence from cross-sectional and longitudinal studies about the associations between brain pathologies and late-life BMI.^45,46,47^ Previous studies showed that a greater burden of AD pathology was associated with lower BMI.^45,46^ One longitudinal study^46^ based on the Alzheimer Disease Neuroimaging Initiative (ADNI) with positron emission tomography imaging reported that AD biomarkers were not associated with BMI change (up to 2 years follow-up) regardless of cognitive function. In contrast, the other one based on the Harvard Aging Brain Study and ADNI found that a higher AD burden was associated with a significantly faster decline in BMI over a median of more than 4 years among cognitively healthy older adults.^47^ The discrepancies in previous studies may be due to methodological issues (eg, varied sample size and follow-up period). Several studies using brain pathology data obtained via autopsy have reported an association between higher burdens of AD pathologies and a lower BMI proximate to death.^29,48,49^ However, previous studies reported no significant association between BMI and cerebral vascular pathologies.^29,49^ Rather than using average BMI across follow-ups to evaluate BMI change, as was the case in previous studies^29,48^ we conducted the mixed-effect model analysis with a backward timescale, accounting for variability of BMI. We found that BMI was associated with a more steep declination in those with a high (vs low) burden of AD pathology or cerebral vascular disease pathology among dementia-free participants. Our findings suggest that the high levels of AD pathology or cerebral vascular disease pathology may be associated with the BMI decline preceding MCI. Future imaging studies (eg, using positron emission tomography) are warranted to clarify the temporal association between BMI change and brain pathologies.

### Strengths and Limitations

Strengths of this study include the community-based cohort study with a relatively large sample, a long-term follow-up, annually repeated BMI measurements, clinical diagnoses of MCI and dementia following standard criteria, and the availability of brain autopsy data. However, some limitations need to be pointed out. First, participants in MAP were volunteers from the community, had a high level of education, and performed well on cognitive tests. However, the characteristics of MAP participants are generally similar to those in other well-established cohorts such as the Honolulu-Asia Aging Study^50^ and the Kungsholmen Project^51^ in terms of demographics and age at MCI and dementia onset. Nevertheless, caution is needed when generalizing our findings to other populations, especially to younger-old adults (eg, 75 years). Second, there may be selection bias because we only included participants with at least 3 BMI measurements. Still, we conducted sensitivity analyses including all participants with at least 1 BMI measurement, and the trajectories were largely unchanged. Third, power may be limited when investigating BMI differences at some time points with the relatively few observations. Future population-based longitudinal studies with a large sample size and long follow-up are warranted to verify our findings. Fourth, as data on brain pathologies were obtained via brain autopsy after the participant’s death, we have no information on when exactly the brain pathologies developed and therefore cannot conclude causal associations. Future studies using longitudinal AD biomarkers data are warranted to clarify the associations between AD burden and weight change. Finally, an accelerated weight loss may occur near the end of life.^52^ However, the BMI trajectories did not change substantially when we restricted the analysis to only participants with BMI measurements taken at least two years before death.

## Conclusions

This cohort study provides evidence on the long-term BMI decline before the development of MCI and the progression of MCI to dementia. Compared with cognitively intact individuals, those who develop MCI may experience a significant associated BMI decline earlier and have a lower BMI starting from approximately 7 years before MCI diagnosis. BMI continues to decline after MCI diagnosis at the same pace in people who develop dementia and those who do not. We also found that AD and cerebral vascular disease pathology might explain the BMI decline in the lead-up to the development of cognitive impairment. These findings highlight the importance of monitoring weight change regularly among older adults, as a faster decline in BMI and a lower BMI may indicate the need for cognitive assessments. Monitoring weight change may help to identify high-risk populations in the early stage of MCI for early intervention to prevent or delay dementia onset. Further studies are needed to confirm our findings and better elucidate the mechanisms underlying the BMI change during the preclinical and prodromal phase of dementia.

## Notes

Supplement. **eAppendix 1.** Data Collection **eAppendix 2.** Supplementary Analyses **eFigure 1.** Flowchart of the Study Population **eFigure 2.** Trajectories of Body Mass Index (BMI) in the 18 Years Before Mild Cognitive Impairment (MCI) Diagnosis Excluding the BMI Measured Within 2 Years Before Death **eFigure 3.** Trajectories of Body Mass Index (BMI) in the 10 Years Before Dementia Diagnosis Among Participants With Incident Mild Cognitive Impairment (MCI) Excluding the BMI Measured Within 2 Years Before Death **eTable 1.** β Coefficient and 95% CI of Body Mass Index (BMI) Trajectories Before Mild Cognitive Impairment (MCI) **eTable 2.** β Coefficient and 95% CI of the Association Between Dementia and Body Mass Index (BMI) Changes Before Diagnosis of Dementia Among Participants With Incident Mild Cognitive Impairment (MCI) **eTable 3.** β Coefficient and 95% CI of the Association Between Dementia and Body Mass Index (BMI) Trajectories With Brain Pathology Before Death Among Participants Without Incident Dementia **eTable 4.** β Coefficient and 95% CI of the Association Between Mild Cognitive Impairment (MCI) and Body Mass Index (BMI) Change Before Diagnosis, With a Break Point at Year −7 **eTable 5.** Odds Ratio (OR) and 95% CI of the Incident Mild Cognitive Impairment (MCI) According to Body Mass Index (BMI) Change 18 to 7 Years Before the Diagnosis of MCI or End of Follow-up
eReferences
