# Supplementary material for: Body Mass Index Trajectories Preceding Incident Mild Cognitive Impairment and Dementia
Source: JAMA Psychiatry. 2022 Oct 26;79(12):1180–7. doi: 10.1001/jamapsychiatry.2022.3446 (PMC9608028; doi:10.1001/jamapsychiatry.2022.3446)
Supplement: Supplement. — eAppendix 1. Data Collection eAppendix 2. Supplementary Analyses eFigure 1. Flowchart of the Study Population eFigure 2. Trajectories of Body Mass Index (BMI) in the 18 Years Before Mild Cognitive Impairment (MCI) Diagnosis Excluding the BMI Measured Within 2 Years Before Death eFigure 3. Trajectories of Body Mass Index (BMI) in the 10 Years Before Dementia Diagnosis Among Participants With Incident Mild Cognitive Impairment (MCI) Excluding the BMI Measured Within 2 Years Before Death eTable 1. β Coefficient and 95% CI of Body Mass Index (BMI) Trajectories Before Mild Cognitive Impairment (MCI) eTable 2. β Coefficient and 95% CI of the Association Between Dementia and Body Mass Index (BMI) Changes Before Diagnosis of Dementia Among Participants With Incident Mild Cognitive Impairment (MCI) eTable 3. β Coefficient and 95% CI of the Association Between Dementia and Body Mass Index (BMI) Trajectories With Brain Pathology Before Death Among Participants Without Incident Dementia eTable 4. β Coefficient and 95% CI of the Association Between Mild Cognitive Impairment (MCI) and Body Mass Index (BMI) Change Before Diagnosis, With a Break Point at Year −7 eTable 5. Odds Ratio (OR) and 95% CI of the Incident Mild Cognitive Impairment (MCI) According to Body Mass Index (BMI) Change 18 to 7 Years Before the Diagnosis of MCI or End of Follow-up eReferences [file jamapsychiatry-e223446-s001.pdf]

Supplementary Online Content

Guo J, Wang J, Dove A, et al. Body mass index trajectories preceding incident mild cognitive impairment and dementia. *JAMA Psychiatry*. Published online October 26, 2022. doi:10.1001/jamapsychiatry.2022.3446

eAppendix 1. Data Collection

eAppendix 2. Supplementary Analyses

eFigure 1. Flowchart of the Study Population

eFigure 2. Trajectories of Body Mass Index (BMI) in the 18 Years Before Mild Cognitive Impairment (MCI) Diagnosis Excluding the BMI Measured Within 2 Years Before Death

eFigure 3. Trajectories of Body Mass Index (BMI) in the 10 Years Before Dementia Diagnosis Among Participants With Incident Mild Cognitive Impairment (MCI) Excluding the BMI Measured Within 2 Years Before Death

eTable 1. β Coefficient and 95% CI of Body Mass Index (BMI) Trajectories Before Mild Cognitive Impairment (MCI)

eTable 2. β Coefficient and 95% CI of the Association Between Dementia and Body Mass Index (BMI) Changes Before Diagnosis of Dementia Among Participants With Incident Mild Cognitive Impairment (MCI)

eTable 3. β Coefficient and 95% CI of the Association Between Dementia and Body Mass Index (BMI) Trajectories With Brain Pathology Before Death Among Participants Without Incident Dementia

eTable 4. β Coefficient and 95% CI of the Association Between Mild Cognitive Impairment (MCI) and Body Mass Index (BMI) Change Before Diagnosis, With a Break Point at Year −7

eTable 5. Odds Ratio (OR) and 95% CI of the Incident Mild Cognitive Impairment (MCI) According to Body Mass Index (BMI) Change 18 to 7 Years Before the Diagnosis of MCI or End of Follow-up

eReferences

This supplementary material has been provided by the authors to give readers additional information about their work.

© 2022 Guo J et al. *JAMA Psychiatry.*

eAppendix 1. Data Collection

Race and ethnicity were categorized as American Indian or Alaska Native, Asian, Black or African American, Native Hawaiian or Other Pacific Islander, White, other, or unknown. Education was recorded as the number of years of formal schooling. Smoking status was categorized as never, former, or current smoking based on self-report. Alcohol consumption was presented as the average amount of alcohol (in grams) consumed per day during the past year. Physical activity was expressed as the total number of hours per week that participants engaged in five types of activity (i.e., walking for exercise, gardening or yard work, calisthenics or general exercise, bicycle riding, and swimming or water exercise) over the past two weeks using questions adapted from the National Health Interview Survey.[1]

Diabetes was ascertained based on the self-reported medical history, glycated haemoglobin (HbA_{1c}) ≥6.5%, fasting plasma glucose ≥126 mg/dl, random blood glucose ≥200 mg/dl, or the use of glucose-lowering medication.[2] Information on vascular diseases – including stroke, claudication, heart conditions (i.e., heart attack or coronary, coronary thrombosis, coronary occlusion, or myocardial infarction), and congestive heart failure – was collected based on self-report. In addition to self-report, evaluation of stroke was also based on neurological exam (when available), cognitive testing, and a physician's interview.

Blood samples were collected for all participants at study entry. Apolipoprotein E (*APOE*) was genotyped by Polymorphic DNA Technologies and participants were dichotomized as carriers vs. non-carriers of the ε4 allele. Further details about the data collection are available on the Rush Alzheimer's Disease Center Resource Sharing Hub at www.radc.rush.edu.

© 2022 Guo J et al. *JAMA Psychiatry.*

eAppendix 2. Supplementary Analyses

We used a piecewise linear mixed-effect model with a breakpoint at year -7 preceding MCI diagnosis. The breakpoint was chosen based on the non-linear trajectory analysis, in which the BMI slope equalled nearly 0 (i.e., turning point) among cognitively intact participants at year -7. Next, we used a linear mixed-effect model to estimate the individual-specific slopes of BMI from year -18 to -7 and from -7 to 0 separately. A logistic regression model was then used to calculate the odds ratios (ORs) for the association between BMI change and the risk of MCI. BMI change was assessed as both a continuous (kg/m^2 per 5 years) and categorical (in tertiles) exposure. The basic-adjusted model included age at year 0, sex, and education as covariates. The multi-adjusted model was further adjusted for smoking status, alcohol consumption, physical activity, vascular diseases, diabetes, and *APOE* ε4. Moreover, we repeated the BMI trajectory analysis after 1) excluding BMI measurements taken within two years of death (i.e., including 5840 observations for BMI trajectories before MCI diagnosis and 1092 observations for BMI trajectories during MCI's progression to dementia) to avoid the effect of pre-death BMI reduction [3] and 2) additionally including participants with at least one BMI measurement (rather than ≥3 measurements) (i.e., including 7426 observations for BMI trajectories before MCI diagnosis and 2075 observations for BMI trajectories during MCI's progression to dementia).

© 2022 Guo J et al. *JAMA Psychiatry.*

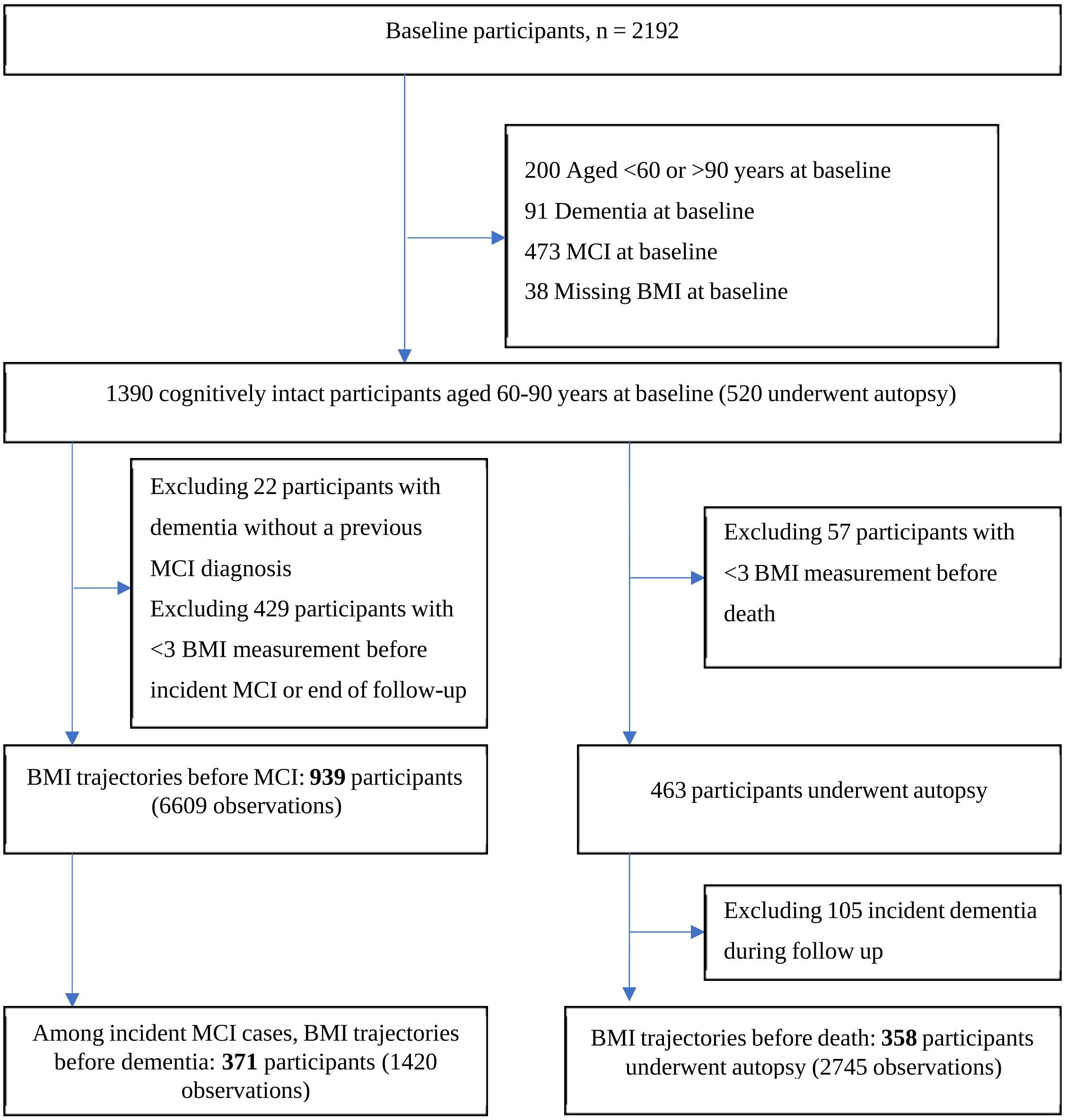

eFigure 1. Flowchart of the Study Population

© 2022 Guo J et al. *JAMA Psychiatry.*

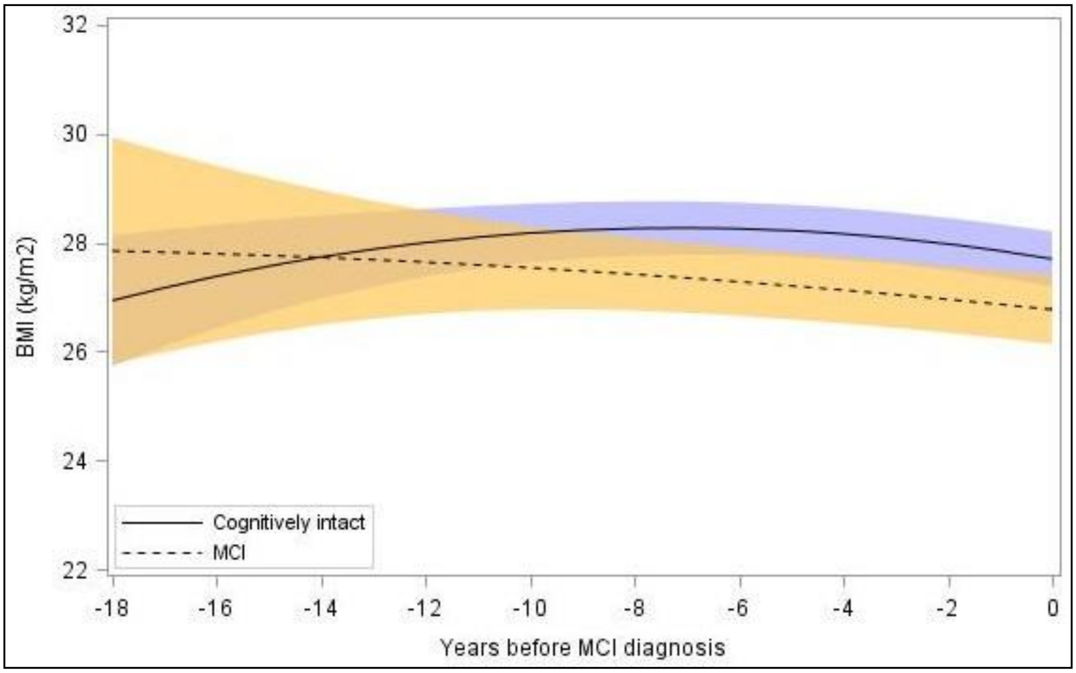

eFigure 2. Trajectories of Body Mass Index (BMI) in the 18 Years Before Mild Cognitive Impairment (MCI) Diagnosis Excluding the BMI Measured Within 2 Years Before Death Diagnosis Among Participants With Incident Mild Cognitive Impairment (MCI) Excluding the BMI Measured Within 2 Years Before Death

The figure represents marginal effects of MCI on trajectories of BMI, adjusted for age at time 0, sex, and education. The band represents the 95% confidence interval of estimated mean BMI.

© 2022 Guo J et al. *JAMA Psychiatry.*

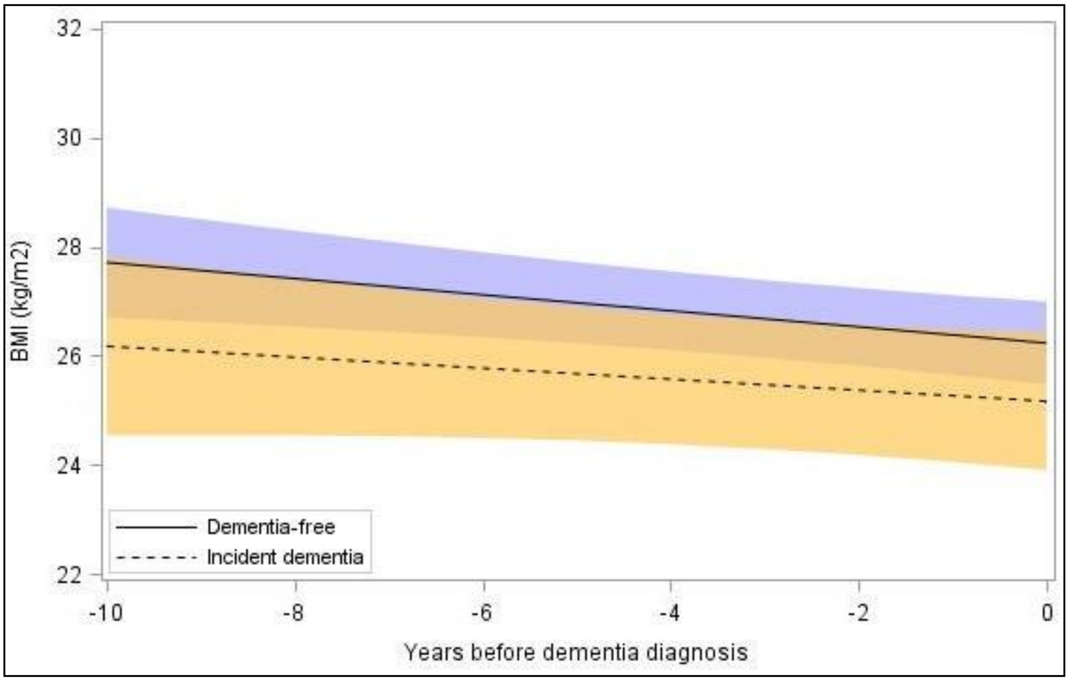

eFigure 3. Trajectories of Body Mass Index (BMI) in the 10 Years Before Dementia

The figure represents marginal effects of dementia on trajectories of BMI, adjusted for age at time 0, sex, and education. The band represents the 95% confidence interval of estimated mean BMI.

© 2022 Guo J et al. *JAMA Psychiatry.*

eTable 1. β Coefficient and 95% CI of Body Mass Index (BMI) Trajectories Before Mild Cognitive Impairment (MCI)

Model terms	β coefficient (95% CI)	*P* value
Intercept	45.32 (40.6, 50.03)	<.001
MCI	-1.07 (-1.77, -0.37)	.003
Time	-0.06 (-0.10, -0.03)	.001
$Time^2$	-0.01 (-0.02, -0.01)	<.001
MCI * Time	-0.03 (-0.09, 0.03)	.32
MCI * $Time^2$	0.01 (0.002, 0.02)	.02

Model adjusted for age at time 0, sex, and education.

© 2022 Guo J et al. *JAMA Psychiatry.*

eTable 2. β Coefficient and 95% CI of the Association Between Dementia and Body Mass Index (BMI) Changes Before Diagnosis of Dementia Among Participants With Incident Mild Cognitive Impairment (MCI)

	Basic-adjusted β coefficient (95% CI)	*P* value		Multi-adjusted β coefficient (95% CI)	*P* value
Intercept					
Dementia-free	41.73 (34.26, 49.20)	<.001		42.60 (34.85, 50.35)	<.001
Incident dementia	41.11 (33.42, 48.79)	<.001		42.14 (34.16, 50.13)	<.001
Difference (Incident dementia vs. dementia-free)	-0.62 (-1.78, 0.53)	.29		-0.45 (-1.62, 0.72)	.45
Slope					
Dementia-free	-0.17 (-0.32, -0.02)	.03		-0.16 (-0.32, -0.01)	.04
Incident dementia	-0.20 (-0.30, -0.10)	<.001		-0.21 (-0.31, -0.10)	<.001
Difference (Incident dementia vs. dementia-free)	-0.03 (-0.21, 0.15)	.73		-0.04 (-0.23, 0.14)	.63

In the basic-adjusted model, we adjusted for age at time 0, sex, and education. In the multi-adjusted model, we further adjusted for smoking status, alcohol consumption, physical activity, vascular diseases, diabetes, and apolipoprotein E ε4.

© 2022 Guo J et al. *JAMA Psychiatry.*

eTable 3. β Coefficient and 95% CI of the Association Between Dementia and Body Mass Index (BMI) Trajectories With Brain Pathology Before Death Among Participants Without Incident Dementia

Model terms	β coefficient (95% CI)	*P* value
For global AD pathology		
Intercept	54.5 (46.06, 62.95)	<.001
Pathology (T2 vs. T1)	-0.14 (-1.37, 1.09)	.83
Pathology (T3 vs. T1)	0.17 (-1.13, 1.46)	.80
Time	-0.05 (-0.13, 0.03)	.20
$Time^2$	-0.23 (-0.37, -0.09)	.001
Pathology * Time (T2 vs. T1)	-0.07 (-0.18, 0.04)	.23
Pathology * Time (T3 vs. T1)	-0.14 (-0.26, -0.02)	.02
Pathology * $Time^2$ (T2 vs. T1)	0.00 (-0.02, 0.02)	.70
Pathology * $Time^2$ (T3 vs. T1)	0.00 (-0.02, 0.02)	.97
For cerebral vascular disease pathology		
Intercept	54.05 (45.69, 62.4)	<.001
Pathology (T2 vs. T1)	-0.98 (-2.12, 0.16)	.09
Pathology (T3 vs. T1)	-1.58 (-2.95, -0.21)	.02
Time	-0.12 (-0.20, -0.04)	.002
$Time^2$	-0.20 (-0.34, -0.07)	.004
Pathology * Time (T2 vs. T1)	-0.004 (-0.11, 0.10)	.94
Pathology * Time (T3 vs. T1)	-0.01 (-0.14, 0.12)	.92
Pathology * $Time^2$ (T2 vs. T1)	0.02 (-0.002, 0.04)	.08
Pathology * $Time^2$ (T3 vs. T1)	0.02 (0.002, 0.05)	.03

T1: lowest tertile, i.e., lowest burden of global AD pathology; T2: middle tertile; T3: highest tertile. Model was adjusted for age at time 0, sex, and education.

© 2022 Guo J et al. *JAMA Psychiatry.*

eTable 4. β Coefficient and 95% CI of the Association Between Mild Cognitive Impairment (MCI) and Body Mass Index (BMI) Change Before Diagnosis, With a Break Point at Year −7

	Basic-adjusted β coefficient (95% CI)	*P* value		Multi-adjusted β coefficient (95% CI)	*P* value
Intercept					
Cognitively intact	43.26 (38.37, 48.15)	<.001		43.99 (38.95, 49.02)	<.001
MCI	45.88 (41.1, 50.65)	<.001		46.49 (41.55, 51.44)	<.001
Difference (MCI vs. cognitively intact)	2.62 (1.5, 3.73)	<.001		2.51 (1.4, 3.62)	<.001
Slope from years -18 to -7					
Cognitively intact	0.07 (0.01, 0.13)	.02		0.07 (0.01, 0.14)	.02
MCI	-0.14 (-0.25, -0.03)	.01		-0.13 (-0.24, -0.03)	.02
Difference (MCI vs. cognitively intact)	-0.22 (-0.34, -0.09)	<.001		-0.21 (-0.33, -0.08)	.001
Slope from years -7 to 0					
Cognitively intact	-0.11 (-0.15, -0.08)	<.001		-0.11 (-0.14, -0.07)	<.001
MCI	-0.09 (-0.14, -0.04)	<.001		-0.09 (-0.14, -0.04)	<.001
Difference (MCI vs. cognitively intact)	0.02 (-0.04, 0.08)	.78		0.02 (-0.04, 0.08)	.55
Slope change between -18 to -7 years and -7 to 0 years					
Cognitively intact	-0.19 (-0.25, -0.12)	<.001		-0.18 (-0.25, -0.12)	<.001
MCI	0.05 (-0.06, 0.16)	.39		0.05 (-0.07, 0.16)	.42
Difference (MCI vs. cognitively intact)	0.24 (0.10, 0.37)	<.001		0.23 (0.10, 0.36)	<.001

In the basic-adjusted model, we adjusted for age at time 0, sex, and education. In the multi-adjusted model, we further adjusted for smoking status, alcohol consumption, physical activity, vascular diseases, diabetes, and apolipoprotein E ε4.

© 2022 Guo J et al. *JAMA Psychiatry.*

eTable 5. Odds Ratio (OR) and 95% CI of the Incident Mild Cognitive Impairment (MCI) According to Body Mass Index (BMI) Change 18 to 7 Years Before the Diagnosis of MCI or End of Follow-up

BMI change (kg/m^2/y)	No. of subjects	No. of MCI	OR (95% CI)	
			Basic-adjusted model [a]	Multi-adjusted model [b]
Continuous [c]	313	97	1.60 (1.10, 2.32)	1.61 (1.10, 2.37)
Categories				
T1 (≥-0.481, <-0.045)	103	39	1.83 (1.04, 3.25)	1.76 (0.97, 3.20)
T2 (≥-0.045, <0.028)	103	35	1.61 (0.91, 2.86)	1.79 (0.99, 3.24)
T3 (≥0.028, <0.786)	107	23	Reference	Reference

Abbreviations: T1: lowest tertile, i.e., fastest BMI decline; T2: middle tertile; T3: highest tertile.

[a] Basic-adjusted model included age at year 0, sex, and education.

[b] Multi-adjusted model included age at year 0, sex, education, smoking status, alcohol consumption, physical activity, vascular diseases, diabetes, and apolipoprotein E ε4.

[c] The estimate is corresponding to per 1 kg/m^2 decrease per 5-year.

© 2022 Guo J et al. *JAMA Psychiatry.*

eReferences

1. 1985 Health Interview Survey. National Center for Health Statistics, Series 10. Publication No. 160 PHHS (PHS). Hyattsville, MD: US Public Health Service; 1985;86–1568
2. Association AD. 2. Classification and Diagnosis of Diabetes: Standards of Medical Care in Diabetes—2021. Diabetes Care. 2021;44(Supplement_1):S15-S33. doi:10.2337/dc21-S002.
3. Alley DE, Metter EJ, Griswold ME, et al. Changes in Weight at the End of Life: Characterizing Weight Loss by Time to Death in a Cohort Study of Older Men. Am J Epidemiol. 2010;172(5):558-565. doi:10.1093/aje/kwq168.

© 2022 Guo J et al. *JAMA Psychiatry.*
